# Exposure to Early Life Stress Results in Epigenetic Changes in Neurotrophic Factor Gene Expression in a Parkinsonian Rat Model

**DOI:** 10.1155/2016/6438783

**Published:** 2016-01-03

**Authors:** Thabisile Mpofana, Willie M. U. Daniels, Musa V. Mabandla

**Affiliations:** Discipline of Human Physiology, School of Laboratory Medicine and Medical Sciences, University of KwaZulu-Natal, Westville Campus, Durban 4000, South Africa

## Abstract

Early life adversity increases the risk of mental disorders later in life. Chronic early life stress may alter neurotrophic factor gene expression including those for brain derived neurotrophic factor (BDNF) and glial cell derived neurotrophic factor (GDNF) that are important in neuronal growth, survival, and maintenance. Maternal separation was used in this study to model early life stress. Following unilateral injection of a mild dose of 6-hydroxydopamine (6-OHDA), we measured corticosterone (CORT) in the blood and striatum of stressed and nonstressed rats; we also measured DNA methylation and BDNF and GDNF gene expression in the striatum using real time PCR. In the presence of stress, we found that there was increased corticosterone concentration in both blood and striatal tissue. Further to this, we found higher DNA methylation and decreased neurotrophic factor gene expression. 6-OHDA lesion increased neurotrophic factor gene expression in both stressed and nonstressed rats but this increase was higher in the nonstressed rats. Our results suggest that exposure to early postnatal stress increases corticosterone concentration which leads to increased DNA methylation. This effect results in decreased BDNF and GDNF gene expression in the striatum leading to decreased protection against subsequent insults later in life.

## 1. Introduction

Parkinson's disease (PD) is one of the most prevalent neurodegenerative disorders with age being the major risk factor [1]. Parkinson's disease is mostly idiopathic in nature with genetic factors contributing about 1% to its etiology [2]. The idiopathic nature of this debilitating disease is thought to be mainly due to multiple factors caused by the interaction between genetic and environmental factors [3, 4]. Environmental factors have been shown to play a role in the modification of genetic composition by altering epigenetic mechanisms, thus leading to altered gene expression [5]. Epigenetic changes may alter the proper development of the offspring if experienced early in life [6, 7]. The most common epigenetic modifications are DNA methylation and histone modification [8]. DNA methylation is believed to be the key epigenetic modification and a marker and regulator of gene expression [9]. The process of DNA methylation is catalyzed by a family of enzymes known as DNA methyltransferases (Dnmts) that transfer a methyl group from S-adenyl methionine (SAM) to the fifth carbon of a cytosine residue to form a methylated form of the DNA base cytosine known as 5-methylcytosine (5-mC) [8].

Early life stress has been implicated in some of the most prevalent mental disorders and this has led to increasing interest in the effects of stress on neurodegeneration [10]. Victims of early life adversity have been linked to a greater risk of mental illness [11, 12]. Exposure to stress early in life has been shown to alter the behaviour and physiology in certain brain areas; this is in part a result of alterations in gene expression in these brain areas [13, 14]. Epigenetic changes linked to environmental factors during early development may affect the expression of genes responsible for the stress response [13].

Prolonged exposure to early life stress increases the baseline circulating glucocorticoid levels; this amongst many of its effects has been associated with compromised neuronal survival [15, 16]. Acute and chronic models of stress result in reduced levels of hippocampal brain derived neurotrophic factor (BDNF) [17, 18]. This decrease in BDNF has been associated with depression in humans [19]. BDNF which is the most active member of the neurotrophin family functions primarily to regulate neuronal differentiation, growth, and neuronal plasticity [20]. Glial cell derived neurotrophic factor (GDNF) is an important neuroprotective ligand for midbrain dopaminergic neurons and it has been shown to reverse functional deficits and prevent nigrostriatal neurodegeneration in primate models of PD [21]. It has been shown that GDNF promotes the development and differentiation of dopaminergic neurons [22]. BDNF and GDNF in the striatum are found in small quantities compared to the hippocampus but they play an important role in the development and survival of dopaminergic neurons providing protection against neurodegeneration [20]. The aim of this study was to assess mechanisms by which stress exposes dopamine neurons to insults later in life by investigating the effects of early maternal stress on corticosterone, DNA methylation status, and neurotrophic factor gene expression.

## 2. Materials and Methods

### 2.1. Animals

A total of thirty-six rats were obtained from the Biomedical Research Unit of the University of KwaZulu-Natal and kept under standard lab conditions with food and water freely available. On postnatal day 1 (PND 1), the rats were sexed and culled to 6 male pups per litter. Rats were randomly divided into two equal groups. One group was normally reared (NS) and the second group was maternally separated (MS). The maternal separation protocol started on postnatal day 2 and lasted until postnatal day 14. Rats were further subdivided into 4 groups; group 1 was normally reared saline lesioned rats (NS-control); group 2 was normally reared 6-OHDA lesioned rats (NS), group 3 was maternally separated saline rats (MS-control), and group 4 was maternally separated 6-OHDA lesioned rats (MS). All procedures were approved by the Animal Ethics Committee of the University of KwaZulu-Natal (017/15/Animal).

### 2.2. Maternal Separation

A 3-hour (9 h00 to 12 h00) daily maternal separation protocol was used. Dams were removed from the home cage and placed in a cage with clean bedding. Pups were then removed from the experimental room and placed in a different room to prevent any form of communication between pups and dams. After 3 hours the pups were returned to the animal room and dams were placed back in the home cage. The daily light/dark cycle was from 6 a.m. to 6 p.m. All cages were cleaned once a week. The rats were weaned on postnatal day 21 and left undisturbed until postnatal day 60.

### 2.3. Stereotaxic Surgery

Desipramine (15 mg/kg i.p., Sigma, Munich, Germany), a norepinephrine reuptake blocker which serves to prevent 6-OHDA uptake by noradrenergic neurons, was injected thirty minutes prior to the injection of 6-OHDA. Prior to the injection of 6-OHDA, the animals were anaesthetised with sodium pentobarbital (0.2 mL/kg i.p., Lakato, South Africa). To aid with respiration during unconsciousness, animals received atropine (0.2 mL/kg i.p., Sigma, Munich, Germany) before being placed in the stereotaxic apparatus (David Kopf Instruments, Tujunga, USA). Animals received an injection of 6-OHDA HCl (5 *μ*g/4 *μ*L dissolved in 0.2% ascorbic acid; Sigma, St. Louis, MO, USA) infusion unilaterally (0.5 *μ*L/min) using a Hamilton syringe into the right medial forebrain bundle (4.7 mm anterior to lambda, 1.6 mm lateral to midline, and 8.4 mm ventral to dura) according to the Paxinos and Watson rat brain atlas [23]. These rats were kept in the home room (two rats per cage) for two weeks (until PND 75).

### 2.4. Sacrifice and Neurochemical Analysis

Animals were sacrificed by decapitation on postnatal day 75 using a guillotine. Trunk blood was collected into vacutainer tubes (coated with EDTA to prevent clotting) which were then centrifuged at 3500 rpm for 10 minutes at 4°C in a refrigerated centrifuge (Z326, Lasec, South Africa). Plasma was transferred into Eppendorfs and quickly frozen in liquid nitrogen. Immediately after decapitation, the brain was removed from the skull and placed in frozen 0.9% saline slush before the striatum was dissected out and quickly frozen in liquid nitrogen. The frozen tissue was stored in a biofreezer at −80°C where it remained until the day of analysis.

### 2.5. Corticosterone ELISA

Corticosterone concentration was measured in blood and striatal tissue using rat corticosterone ELISA kit (Anatech, South Africa). Striatal brain tissue was homogenised in PBS (600 *μ*L) before being used. The sonicated sample, standards and controls (20 *μ*L each), was pipetted into respective well plates. Enzyme conjugate (200 *μ*L) was added into each well followed by incubation (60 minutes). After the incubation period, the wells were washed after which Substrate Solution (100 *μ*L) was added into each well. After 15-minute incubation, Stop Solution (50 *μ*L) was added to each well to stop the reaction. Optical density (OD) was measured at 450 nm within 10 minutes.

### 2.6. DNA Methylation

Tissue analysis included quantification of DNA methylation using 5-mC DNA ELISA (Zymo Research, California, USA). The protocol consisted of extraction and quantification. Extraction continued overnight and quantification was performed the following day.

#### 2.6.1. Tissue Preparation

Frozen striatal tissue was prepared using ZR Genomic DNA-Tissue Mini Prep (Inqaba Biotechnical Industries, South Africa) as follows: a solution consisting of H_2_O (95 *μ*L), 2x digestion buffer (95 *μ*L), and proteinase K (10 *μ*L) was added to tissue containing microcentrifuge tubes. The solution was mixed and incubated for 3 hours at 55°C. After incubation, genomic lysis buffer (700 *μ*L) was added to each tube and mixed by vortexing. The mixture was centrifuged at 10,000 ×g for 1 minute after which the supernatant was transferred to a zymo-spin IIC column in collecting tubes and centrifuged at 10,000 ×g for 1 minute. DNA prewash buffer (200 *μ*L) was added to the spin column in a new collecting tube and centrifuged for 1 minute. g-DNA wash buffer (400 *μ*L) was added to the spin column and then centrifuged for 1 minute. The spin column was then transferred to a clean microcentrifuge tube; DNA elution buffer (200 *μ*L) was added to the spin column followed by 5-minute incubation at room temperature. The tubes were then centrifuged for 30 seconds at 5000 ×g after which they were stored at −20°C.

#### 2.6.2. % 5-mC DNA Methylation

The prepared DNA (5 *μ*L) was added to 5-mC coating buffer (95 *μ*L) in a PCR tube. Standards and negative and positive controls were also prepared (samples were analyzed in triplicate). These were then denatured at 98°C for 5 minutes in a thermal cycler. The denatured DNA was immediately transferred to ice for 10 minutes after which it was transferred to the plate, covered with foil, and incubated at 37°C for 1 hour. Following the incubation, the buffer was discarded and each well was washed 3 times with 5-mC ELISA buffer (200 *μ*L). ELISA buffer (200 *μ*L) was added to each well; the plate was covered and incubated at 37°C for 30 minutes, after which the buffer was discarded. Antibody mix (100 *μ*L) was then added to each well. The plate was covered with foil and incubated at 37°C for 1 hour. For color development, the antibody mix was discarded and each well washed 3 times with 5-mC ELISA buffer (200 *μ*L). HRP developer (100 *μ*L) was added to each well and the plate placed at room temperature for 10–60 minutes to allow for color development. Absorbance was measured at 405 and 450 nm using an ELISA plate reader.

### 2.7. Real Time PCR

RNA was extracted from the tissue using an All ZR RNA MiniPrep Kit (Inqaba Biotechnical Industries, South Africa). Total RNA (1 *μ*g) was reverse-transcribed into cDNA using a cDNA synthesis kit (Bio-Rad, South Africa). cDNA was amplified by real time PCR (Lightcycler 96). The primers (Table 1) were synthesised by Inqaba Biotec, South Africa, with beta actin serving as a reference gene. PCR was performed using Lightcycler 96 consisting of denaturation at 94°C for 5 minutes, 32 additional cycles at 94°C for 50 seconds, and then 45 seconds of primer annealing at 55°C (for BDNF), 55°C (for GDNF), and 59°C (for *β*-actin), followed by final extension at 72°C for 8 minutes.

### 2.8. Statistical Analysis

The data were analyzed using the software program GraphPad Prism (version 5). Nonparametric tests were used followed by Mann-Whitney* U* test where significance was detected. Data are expressed as mean ± SEM. A *P* value < 0.05 was considered significant.

## 3. Results

### 3.1. Brain Corticosterone Concentration

**Figure 1:**
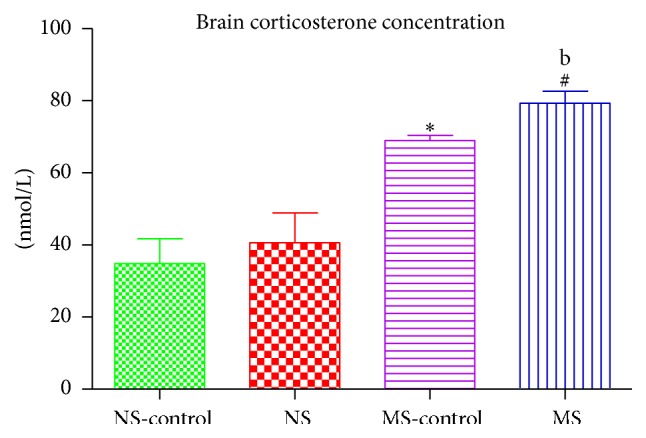
Graph showing striatal corticosterone concentration of nonstressed saline lesioned rats (NS-control), nonstressed rats (NS), maternally separated saline lesioned rats (MS-control), and maternally separated rats (MS) in the lesioned striatum ^*∗*^(NS-control versus MS-control)  *P* < 0.05, ^#^(NS versus MS)  *P* < 0.05, and ^b^(MS-control versus MS)  *P* < 0.05.

Exposure to early postnatal stress increased corticosterone concentration in the striatum of maternally separated rats compared to the normally reared rats: ^*∗*^(NS-control versus MS-control); ^#^(NS versus MS, *P* < 0.05; Figure 1). There was a 6-OHDA effect in striatal corticosterone concentration of lesioned maternally separated rats compared to nonlesioned maternally separated rats ^b^(MS-control versus MS, *P* < 0.05; Figure 1).

### 3.2. Blood Corticosterone Concentration

**Figure 2:**
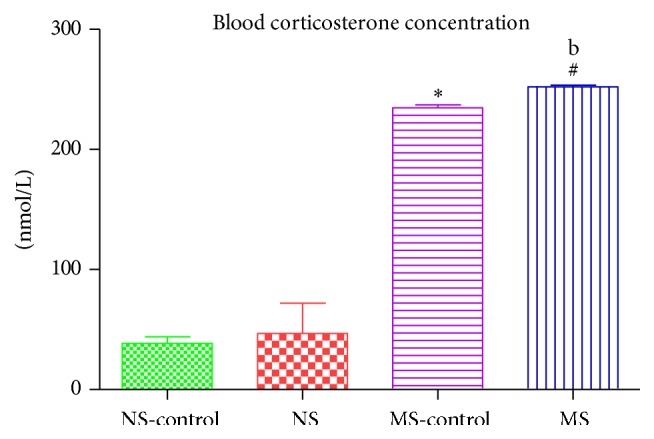
Graph showing blood corticosterone concentration of nonstressed saline lesioned rats (NS-control), nonstressed rats (NS), maternally separated saline lesioned rats (MS-control), and maternally separated rats (MS). ^*∗*^(NS-control versus MS-control)  *P* < 0.05, ^#^(NS versus MS)  *P* < 0.05, and ^b^(MS-control versus MS)  *P* < 0.05.

The effect of postnatal stress led to an increase in plasma corticosterone concentration in stressed rats compared to the nonstressed rats: ^*∗*^(NS-control versus MS-control); ^#^(NS versus MS, *P* < 0.05; Figure 2). There was a 6-OHDA effect on plasma corticosterone concentration of the lesioned maternally separated rats compared to nonlesioned maternally separated rats ^b^(MS-control versus MS, *P* < 0.05; Figure 2).

### 3.3. DNA Methylation in the Striatum

**Figure 3:**
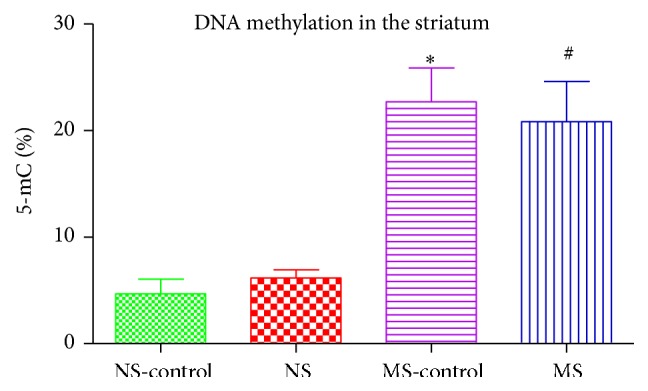
Graph showing % 5-mC in the striatum of nonstressed saline lesioned rats (NS-control), nonstressed rats (NS), maternally separated saline lesioned rats (MS-control), and maternally separated rats (MS): ^*∗*^(NS-control versus MS-control)  *P* < 0.05; ^#^(NS versus MS)  *P* < 0.05.

Exposure to early postnatal stress increased DNA methylation as there was a higher % 5-mC in maternally separated rats compared to the nonstressed rats: ^*∗*^(NS-control versus MS-control); ^#^(NS versus MS, *P* < 0.05; Figure 3).

### 3.4. BDNF Gene Expression

**Figure 4:**
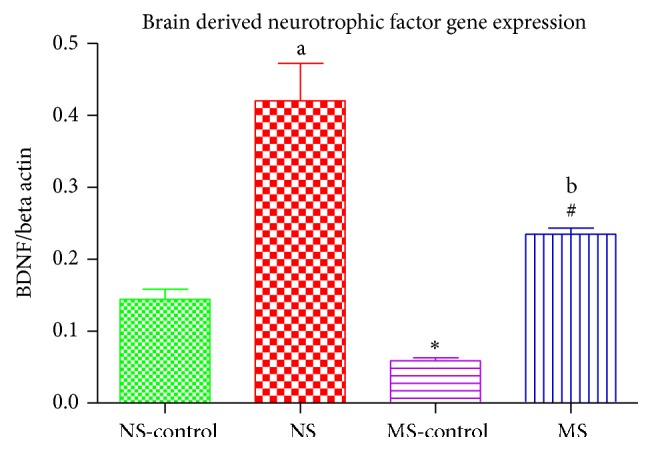
Graph showing BDNF gene expression in the striatum of nonstressed saline lesioned rats (NS-control), nonstressed rats (NS), maternally separated saline lesioned rats (MS-control), and maternally separated rats (MS). ^*∗*^(NS-control versus MS-control)  *P* < 0.05, ^a^(NS-control versus NS)  *P* < 0.05, ^#^(MS-control versus MS)  *P* < 0.05, and ^b^(NS versus MS, *P* < 0.05).

There was a stress effect on BDNF gene expression on maternally separated rats compared to nonstressed rats: ^*∗*^(NS-control versus MS-control, *P* < 0.05; Figure 4). There was a 6-OHDA effect on BDNF gene expression on both stressed and nonstressed rats: ^a^(NS-control versus NS, *P* < 0.05); ^#^(MS-control versus MS, *P* < 0.05; Figure 4). Stress decreased BDNF gene expression in lesioned rats: ^b^(NS versus MS, *P* < 0.05; Figure 4).

### 3.5. GDNF Gene Expression

**Figure 5:**
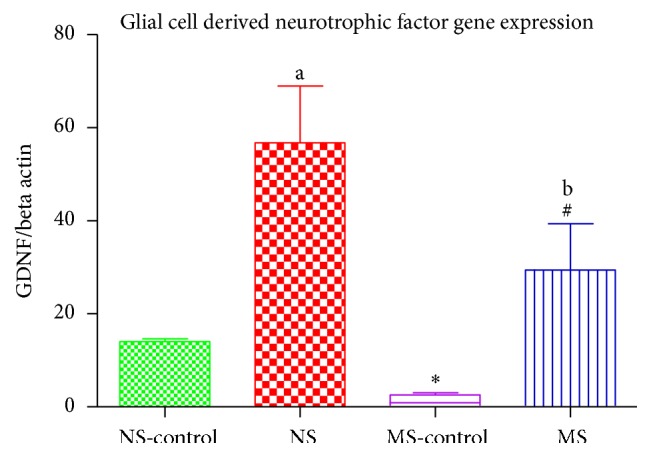
Graph showing GDNF gene expression in the striatum of nonstressed saline lesioned rats (NS-control), nonstressed rats (NS), maternally separated saline lesioned rats (MS-control), and maternally separated rats (MS). ^*∗*^(NS-control versus MS-control)  *P* < 0.05, ^a^(NS-control versus NS)  *P* < 0.05, ^#^(MS-control versus MS)  *P* < 0.05, and ^b^(NS versus MS, *P* < 0.05).

There was a stress effect on neurotrophic factor gene concentration: ^*∗*^(NS-control versus MS-control, *P* < 0.05; Figure 5). There was also a 6-OHDA effect on GDNF gene expression in both stressed and nonstressed rats: ^a^(NS-control versus NS), ^#^(MS-control versus MS, *P* < 0.05; Figure 5). Stress decreased GDNF expression in lesioned rats: ^b^(NS versus MS, *P* < 0.05; Figure 5).

## 4. Discussion

Studies have shown that exposure to early postnatal stress may increase the vulnerability of dopaminergic neurons to degeneration later in life [24]. Our study aimed to investigate mechanisms by which early life stress may exacerbate neurodegeneration. Pups were maternally separated during the stress hyporesponsive period (PND 2–14) to model early postnatal stress. This period is particularly important in the development of the hypothalamic pituitary adrenal (HPA) axis [25]. Prolonged exposure to stress during the stress hyporesponsive period leads to the dysregulation of the HPA axis leading to increased circulating glucocorticoids [25]. We assessed corticosterone concentration as a measure of HPA axis activity in blood and striatal tissue. We found that, in the presence of maternal separation stress, corticosterone concentration was increased in both plasma and brain tissue. The induction of a 6-OHDA lesion increased corticosterone concentration in maternally separated rats compared to nonstressed rats. We injected a subclinical dose of 6-OHDA which is known to produce a mild lesion; this was, however, exacerbated by the presence of maternal separation stress on the postnatally stressed rats. Studies have shown that prolonged early life stress exposes the developing neural circuits to high circulating glucocorticoids with corticosterone being the most secreted stress hormone [15]. Glucocorticoids under normal conditions have important metabolic functions; however prolonged exposure has been shown to alter neuronal development and morphology [13, 14]. These changes have been shown to be long-lasting and may have negative effects on the adult brain [13].

We also measured the percentage of global DNA methylation in the striatal tissue by measuring the percentage of 5-methylcytosine (% 5-mC). We found a higher % 5-mC in the striatum of rats that were exposed to maternal separation stress compared to nonstressed rats. It has been hypothesised that maternal separation leads to behavioural and neuroendocrine changes and may also alter the DNA methylation status [26]. Chronic early life exposure to adversity has enduring effects on the developing brain; these effects may predispose the brain to degenerative disease later in life [25].

Induction of the 6-OHDA lesion did not have an effect in % 5-mC as there were no differences between the nonstressed control and the nonstressed lesioned rats and also no significant changes in the % 5-mC in the maternally separated control rats compared to the maternally separated lesioned rats. This increase seen in the stressed rats suggests that prolonged early maternal stress negatively affects the proper development of the neural circuits altering gene expression, thus facilitating epigenetic changes that will result in modification of genes [27]. Our results show that maternal separation stress during the early postnatal period (PND 2–14) causes epigenetic changes that are long-lasting in the brain, these effects are seen later in life (PND 75), and they render the brain vulnerable to degeneration in the presence of a toxic insult. Although there is some evidence for the role of DNA methylation in Parkinson's disease [28], the 6-OHDA model of Parkinson's disease adopted in our experiments did not affect the percentage global DNA methylation in the striatum. The severity of dopamine degeneration in a Parkinsonian rat model depends on the dose and location of the 6-hydroxydopamine injection [29, 30]. Different doses of 6-OHDA mimic different stages of Parkinsonism with the 5 *μ*g/4 *μ*L dose producing a mild lesion [29]. Parkinson's disease is clinically diagnosed after there has been 80% or more dopamine degeneration [31]. The 6-OHDA dose we injected does not produce a lesion greater than 80% [32] and hence does not present with all dysfunctions associated with Parkinson's disease.

When we measured the expression of neurotrophic factor genes, we found a lower concentration of BDNF and GDNF gene expression in the stressed rats compared to the normally reared rats. The current result suggests that early life adversity alone may cause epigenetic changes that weaken the brain's ability to protect itself against an injury or insult. This is further supported by the lower neurotrophic factor gene expression in the stressed rats that were injected with 6-OHDA. In the presence of 6-OHDA, there was an increase in the neurotrophic factor gene expression on both the nonstressed and stressed rats in response to tissue injury. This increase was, however, much higher in the normally reared rats when compared to the stressed rats. Our results show that stressed rats were not as responsive to the injury as the nonstressed rats. This may be the reason we see greater dopamine degeneration in stressed animals following 6-OHDA injection [33, 34]. These neurotrophic factors are known to play an important role in the growth and survival of neurons [35]. It is known that high corticosterone concentration suppresses the secretion of BDNF in the hippocampus [36–38] and we have gone a step further and shown that, in the presence of high concentration of corticosterone, the expression of neurotrophic factor genes (BDNF and GDNF) is also compromised in the striatum. Therefore corticosterone enhances epigenetic changes in BDNF and GDNF gene expression suggesting that stress interferes with the expression of BDNF and GDNF, thereby decreasing the secretion of these neurotrophic factors. This is of uttermost importance due to the important function of these neurotrophic factors in neuroprotection and neurogenesis.

## 5. Conclusion

Maternal separation is a form of early life stress that has been shown to alter the developing neuronal circuitry. This has been previously linked to the development of neurological disorders.

In agreement with previous studies, our findings have shown that early life stress in the form of maternal separation increases baseline circulating corticosterone concentration leading to the alteration in gene expression. These epigenetic changes result in an altered expression of BDNF and GDNF genes which may lead to a decrease in the concentration of these neurotrophic factors. This results in the brain being more vulnerable to insults leading to exacerbated neurodegeneration.

## Figures and Tables

**Table 1:** The list of primers used in the study.

Gene	Forward	Reverse
GDNF	5′-ACGAAACCAAGGAGGAACTGA-3′	5′-TTTGTCGTACATTGTCTCGGC-3′
BDNF	5′-TCTACGAGACCAAGTGTAATCC-3′	5′-TATGAACCGCCAGCCAAT-3′
